# Targeting the HERV-K102 envelope elicits pyroptosis and represents a novel therapeutic strategy for acute myeloid leukemia

**DOI:** 10.1007/s44313-026-00134-5

**Published:** 2026-04-14

**Authors:** Lihong Zong, Qian Luo, Jingru Zhao, Huyi Lei, Hui Liu, Mengyuan Li, Yiyuan Chen, Minghuan Zhang, Rongzhen Xu, Wenbin Qian

**Affiliations:** 1 Department of Hematology, The Second Affiliated Hospital, College of Medicine, Zhejiang University, Hangzhou, Zhejiang China; 2 Key Laboratory of Cancer Prevention and Intervention, China National Ministry of Education; Biotherapy Research Center, The Second Affiliated Hospital, College of Medicine, Zhejiang University, Hangzhou, China; 3 Department of Hematology, The Affiliated Hospital of ShaoXing University (ShaoXing Municipal Hospital), Shaoxing, Zhejiang China

**Keywords:** HERV-102, AML, Pyroptosis

## Abstract

**Objective:**

Several recent studies have focused on human endogenous retroviruses (HERVs). HERVs entered the human genome millions of years ago and are associated with various diseases including cancer and immune regulation. Among these, the HERV-K family exhibits the highest transcriptional activity. However, little is known about the expression of HERVs in acute myeloid leukemia (AML) and their potential as biomarkers or therapeutic targets. This study primarily investigated the role of HERV-K102 in AML development and explored the underlying mechanisms.

**Methods:**

The expression profiles of HERV K102 in AML and normal samples were analyzed using The Cancer Genome Atlas (TCGA) database and AML cell lines. Knockout models were generated using CRISPR-Cas9-mediated deletion of the HERV-K102 envelope (K-Env). Cell viability and pyroptosis rates were measured using the MTT assay and flow cytometry, respectively. Transcriptome analysis was performed to identify differentially expressed genes and related pathways. Pyroptosis markers were detected using qRT-PCR and western blotting. The role of HERV-K102 in AML was validated using an inducible knockout xenograft tumor model.

**Results:**

HERV-K102 was aberrantly activated and highly expressed in AML cells. K-Env depletion inhibited AML cell proliferation and promoted apoptosis. Furthermore, K-Env knockout induced pyroptosis, as indicated by increased lactate dehydrogenase (LDH) release and enhanced cleavage of caspase-1 and gasdermin D (GSDMD). Transcriptomic and functional analyses demonstrated that this process is mediated by S100A9 upregulation and activation of the NOD-like receptor protein 3 (NLRP3) inflammasome pathway.

**Conclusion:**

Our findings suggest that HERV-K102 Env may play an important role in AML pathogenesis and represents a novel diagnostic and therapeutic target.

**Supplementary Information:**

The online version contains supplementary material available at https://doi.org/10.1007/s44313-026-00134-5.

## Introduction

Human endogenous retroviruses (HERVs) are long-terminal repeat (LTR) retrotransposons that constitute approximately 8% of the human genome. Full-length human HERVs contain four essential viral genes (gag, pro, pol, and env) in their internal regions flanked by two LTR regions [1]. Among the HERV family members, HERV-K102 produces retroviral proteins and assembles complete viral particles. Once inside host cells, these particles can reverse transcribe their RNA genome and integrate the resulting DNA into the host genome, thereby generating new proviral loci that closely resemble the existing HERV-K102 sequences [2–4]. HERV expression is tightly regulated in normal adult tissues but has been reported to be aberrantly activated in cancers, inflammatory diseases, aging, and viral infections [5–8]. HERVs may contribute to tumorigenesis by inducing chromosomal instability, promoting abnormal gene expression through LTR activity, or modulating the immune system through RNA and protein products [9]. Elevated HERV-K expression has been observed in several human cancers, including breast cancer [10, 11], ovarian cancer [12], lymphoma [13], melanoma [14], and acute myeloid leukemia (AML) [15]. HERV-K Env is a promising cancer biomarker that has been proposed as a target for immunotherapy [16].

Programmed cell death pathways, particularly pyroptosis, play crucial roles in tumor immunity and inflammation [17]. Pyroptosis is an inflammatory cell death process mediated by inflammasomes such as NLRP3, which activate caspase-1, cleave gasdermin D (GSDMD), and cause plasma membrane rupture, leading to the release of IL-1β and IL-18 [18]. Previous studies have shown that S100A8 and S100A9 play critical roles in regulating inflammatory responses [19]. Specifically, S100A9-mediated activation of the NLRP3 inflammasome promotes pyroptosis, a lytic and inflammatory form of cell death [20, 21]. It is worth noting that the process of inflammation‐related pyroptosis has emerged as a prospective target for cancer therapy.

This study aimed to investigate the role of HERV-K102 in AML and determine whether the knockout of its envelope protein (K-Env) can induce pyroptosis in AML cells. We explored the molecular mechanisms linking the loss of K-Env to inflammatory cell death. Our findings suggest that HERV-K102 Env may serve as a novel diagnostic marker and therapeutic target for AML.

## Methods

### Cell culture and transfection

Human AML cell lines THP-1, OCI-AML3, KG-1α, MV4-11, and Molm13 were obtained from the American Type Culture Collection (ATCC, Manassas, VA, USA). The cells were cultured in RPMI 1640 medium (Gibco, USA) supplemented with 10% fetal bovine serum (FBS; Gibco, USA) and 1% penicillin/streptomycin (Gibco, USA). Guide RNA sequences for CRISPR-Cas9 were designed using GenScript CRISPR Design Tool. Two oligonucleotides targeting K-Env were then inserted. The first gRNA (#1) sequence was 5'-tattccagtcacattgtaactgg-3,’ and the second gRNA (#2) was 5'-tgacagggttaagatttgcgagg-3.’ Lentiviruses were prepared by packaging plasmids with PMD2G and PAX2 along with the target plasmid containing gRNA, and then transfected into THP-1 and MV4-11 cells. The knockout efficiency was verified by qPCR and western blot at 3–4 days after transfection. In vitro experiments, including flow cytometry, LDH assays, and MTT assays, were conducted 5–7 days after the knockout results were verified.

### Flow cytometry

Flow cytometry was performed using a NovoCyte flow cytometer (ACEA Biosciences Inc.). Commercial antibodies were purchased and the following information was obtained: caspase-1 (FAM-FLICA Caspase 1 YVAD Assay Kit, Cat ICT97#), Annexin V (Annexin V, Cat 640,920), and 7-AAD (Biogems, Cat 61,410–00).

### Lactate dehydrogenase (LDH) release assay

Cells from KO and control groups were collected and seeded evenly in 96-well plates. Cell supernatants were collected and LDH release was measured using an LDH assay kit (MCE, Cat HY-K1090). The absorbance at 490 nm was recorded using a microplate reader.

### Reverse transcription quantitative polymerase chain reaction (RT-qPCR)

Total RNA was extracted from the cells using TRIzol reagent (Invitrogen) according to the manufacturer’s instructions. Total RNA (1 μg) was reverse transcribed using a reverse transcription kit (Takara RR036A), and the resulting cDNA served as the template. Quantitative real-time PCR (qRT-PCR) was performed on a LightCycler 480 system using premix (Takara RR820A) following the manufacturer’s instructions. Relative gene expression levels were calculated using the 2^−△△^Ct method. The primers used in this study are listed in Table 1.

**Table 1:** Specific primers for quantitative qRT-PCR

Gene name	Primer sequence (5′ → 3′)
NLRP3-F	CAATGGGGAGGAGAAGGCGT
NLRP3-R	TCTGAACCCCACTTCGGCTC
Caspase1-F	TGAGCAGCCAGATGGTAGAGC
Caspase1-R	TCACTTCCTGCCCACAGACAT
IL-1β-F	CTCTTCGAGGCACAAGGCAC
IL-1β-R	CAAGTCATCCTCATTGCCACTGT
S100A9-F	TGGCTCCTCGGCTTTGACAGAGT
S100A9-R	TGGGTGCCCCAGCTTCACAGA
GAPDH-F	GCACCGTCAAGGCTGAGAAC
GAPDH-R	GTGGTGAAGACGCCAGTGGA
IL-18-F	TCTTCATTGACCAAGGAAATCGG
IL-18-R	TCCGGGGTGCATTATCTCTAC
TNF-α-F	CCTCTCTCTAATCAGCCCTCTG
TNF-α-R	GAGGACCTGGGAGTAGATGAG
HERV K-Env-F	CTGAGGCAATTGCAGGAGTT
HERV K-Env-R	GCTGTCTCTTCGGAGCTGTT

### Western blotting analysis

Total protein was extracted from cultured cells using the M-PER Mammalian Protein Extraction Reagent (Invitrogen) supplemented with 1% protease and a phosphatase inhibitor cocktail (Invitrogen). After centrifugation at 12,000 rpm for 15 min at 4 °C, protein concentrations were determined using the BCA Protein Detection Kit (Beyotime). Equal amounts of proteins were subjected to sodium dodecyl sulfate–polyacrylamide gel electrophoresis and transferred to polyvinylidene difluoride (PVDF) membranes (Bio-Rad Laboratories, Hercules, CA, USA). The membranes were incubated with commercial primary antibodies and visualized using the Tano 5200 Chemiluminescent Imaging System.

The commercial antibodies used in the western blotting assay were as follows: GAPDH (60,004–1-Ig, Proteintech), IL-1β (16,806–1-AP, Proteintech), S100A9 (26,992–1-AP, Proteintech), Caspase-1/P20 (22,915–1-AP, Proteintech), NLRP3 (27,458–1-AP, Proteintech), Cas9 (26,758–1-AP, Proteintech), and GSDMD (AF4012**,** Affinity). HERV-K102 Env protein was detected as previously described [22].

### RNA-Seq data analysis

Raw sequencing data were filtered using Fastp (v0.23.2), low-quality reads were discarded, and the adaptor sequences were trimmed. Clean reads were further processed using in-house scripts to eliminate the duplication bias introduced during library preparation and sequencing. Briefly, clean reads were clustered according to their UMI sequences, with reads sharing the same UMI grouped into one cluster. Reads within each cluster were compared using pairwise alignment, and those with over 95% sequence identity were extracted into subclusters. Consensus sequences for each subcluster were generated through multiple sequence alignments, thereby eliminating errors and biases introduced by PCR amplification or sequencing. Sequences were then used for standard mRNA-seq analysis and mapped to the reference genome using the STAR software (version 2.7.6a). Gene- and transcript-level counts were obtained using featureCounts (Subread-1.5.1; Bioconductor) and expression levels were calculated. Differentially expressed genes and transcripts were identified using the edgeR package (version 3.40.2) with a p-value cutoff of 0.05 and a fold-change threshold of 2. Gene ontology (GO) and Kyoto encyclopedia of genes and genomes (KEGG) enrichment analyses for differentially expressed genes were performed using R (version 4.0) with a p-value cutoff of 0.05.

### Cell viability and apoptosis assays

Cells were seeded in 96-well plates at 5,000 cells per well. Twenty microliters of thiazolyl blue tetrazolium bromide (MTT; Sangon Biotech, China) were added to each well at designated time points. Cells were incubated with MTT for 4 h and then dissolved overnight in a buffer containing 10% sodium dodecyl sulfate (SDS), 5% isobutanol, and 0.012 M HCl. After the formazan crystals dissolved, the absorbance was measured at 562 nm using a microplate reader, and cell growth curves were generated using GraphPad Prism 10.1.

### Statistical analysis

All experimental data were analyzed using GraphPad Prism 10.1. If the data followed a normal distribution with a homogeneity of variance, a t-test was performed; otherwise, a two-sided nonparametric Wilcoxon rank-sum test was performed. Statistical significance was defined as *p* < 0.05.

### Animals

An optimized in-house MV4-11 model was used. Female NOD.Cg-Prkdc^scid^ IL2rgtm^1Wjl^/SzJ (NSG) mice aged 6–8 weeks were intravenously inoculated with 1 × 10^6^ MV4-11-Cas9 or MV4-11-Cas9-K-Env KO cells. One week later, mice were administered doxycycline (Dox) (2 mg/kg, day 0–5) to induce K-Env knockout. The tumor burden was monitored using an IVIS Lumina LT Series III in vivo imaging system (Perkin Elmer, USA).

## Results

### HERV-K102 is highly expressed in AML

**Fig. 1:**
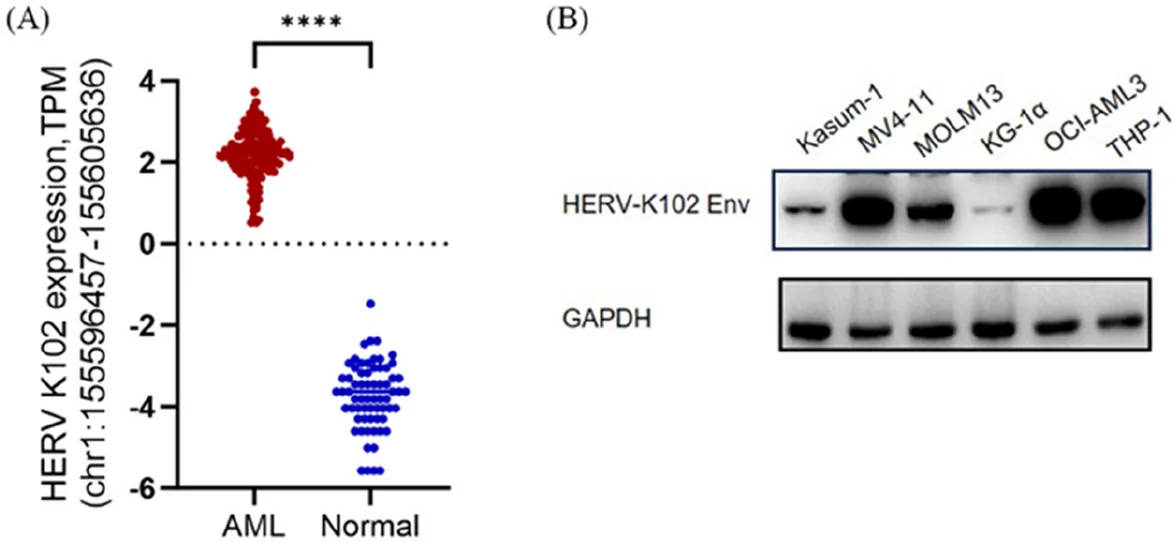
HERV-K102 expression in AML. **A** Expression of HERV-K102 in transcripts per million (TPM) in pan-tissue TCGA and genotype tissue expression (GTEx) datasets. **B** Expression of the HERV-K102 envelope (K-Env) in AML cell lines detected by western blot

To investigate the expression of HERV-K102 in AML, we compared datasets from the pan-tissue TCGA and Genotype-Tissue Expression (GTEx) projects. The expression profile of HERV-K102 was downloaded from an online database (https://xena.ucsc.edu/) based on its chromosomal location (chr1:155,596,457–155,605,636) [2]. HERV-K102 was found to be highly expressed in AML cells (Fig. 1A). The prognostic significance of HERV-K102 was assessed using Kaplan–Meier analysis. As shown in Figure S1, HERV-K102 expression was not significantly associated with overall survival. We then examined K-Env expression in different AML cell lines and observed heterogeneous expression levels. K-Env was highly expressed in OCI-AML3, THP-1, and MV4-11 cells, but showed extremely low expression in KG-1α cells (Fig. 1B).

### K-Env knockout exerts anti-leukemic effects in vitro

**Fig. 2:**
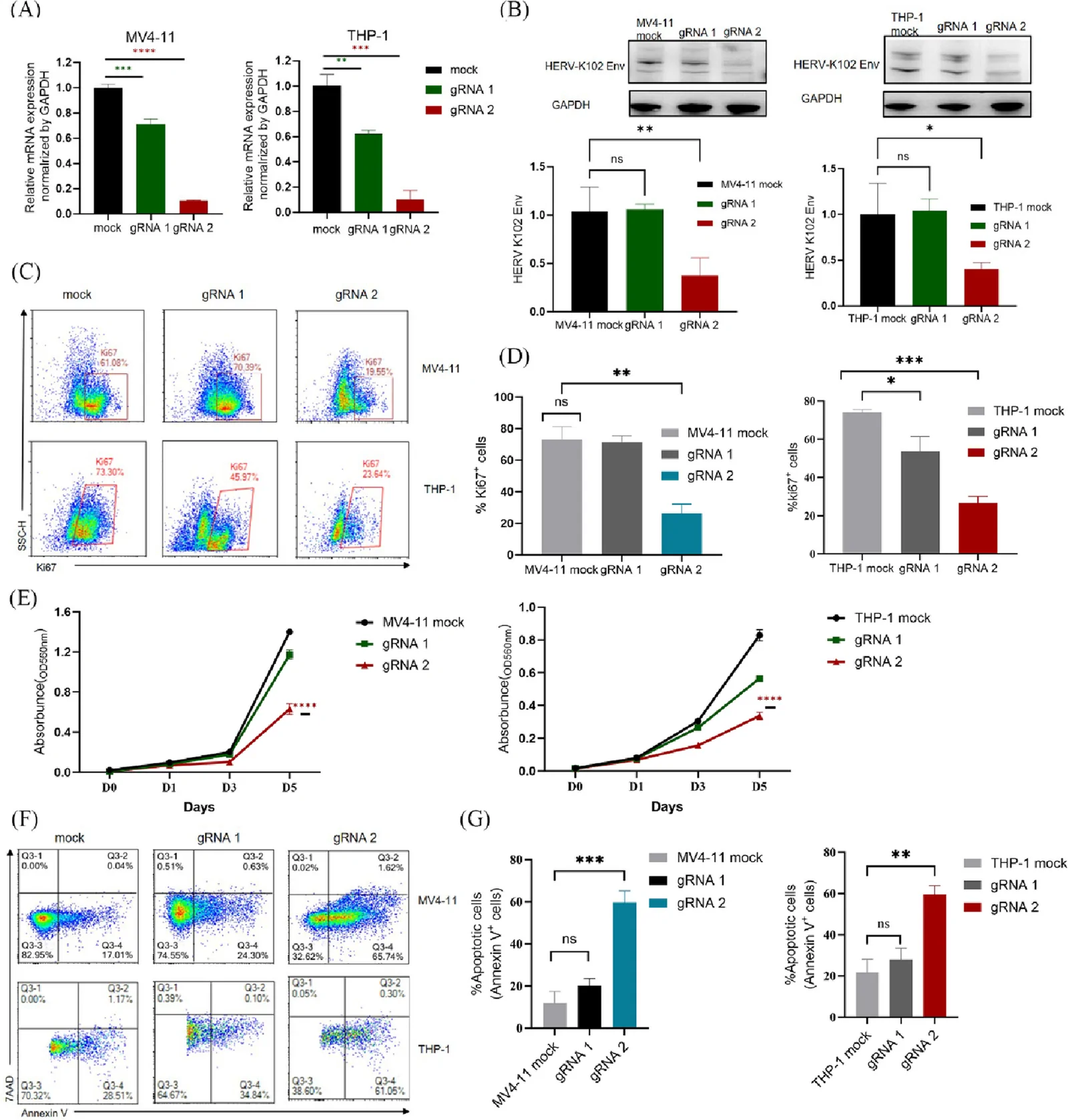
Knockout of K-Env and its effects on AML cells. **A** Quantitative real-time PCR (qRT-PCR) analysis of HERV-K102 mRNA expression in AML cell lines treated with mock, gRNA1, and gRNA2. **B** Representative Western blot images showing the levels of K-Env protein in cells of each treatment group, along with corresponding bar charts. **C**–**D** Flow cytometry assay results showing the proliferation levels in different groups and the bar charts used for visualization. **E** Cell viability determined by MTT assay in three groups. **F**–**G** Flow cytometry assay results showing the apoptosis levels in different groups and bar charts used for visualization

To further investigate the role of K-Env in AML, it was knocked out using the CRISPR-Cas9 gene editing system. Two guide RNAs (gRNAs) targeting K-Env were designed; their specific sequences are described in the Methods section. Here, we selected two cell lines, THP-1 and MV4-11, with high K-Env expression for subsequent experiments. KO efficiency was evaluated by qRT-PCR, and comparative analysis revealed that gRNA2 achieved higher KO efficiency than gRNA1 (Fig. 2A). Western blot analysis confirmed the reduction in HERV-K-Env protein levels (Fig. 2B). Next, we examined whether K-Env knockout affects the survival of leukemia cells. The knockout of K-Env led to a significant decrease in Ki67 expression levels, indicating that cell proliferation was inhibited (Fig. 2C,D). The MTT assay showed that the knockout of K-Env by gRNA2 significantly suppressed the proliferation of leukemia cells (Fig. 2E). Apoptosis was further evaluated using Annexin V and 7-AAD staining, followed by flow cytometry. K-Env knockout by gRNA2 resulted in 59.63% and 60.03% of Annexin V-positive MV4-11 and THP-1 cells, respectively, suggesting the induction of early apoptosis (Fig. 2F,G).

### Transcriptome analysis of HERV-K102 Env KO AML cells reveals activation of pro-inflammatory and immune signaling pathways

**Fig. 3:**
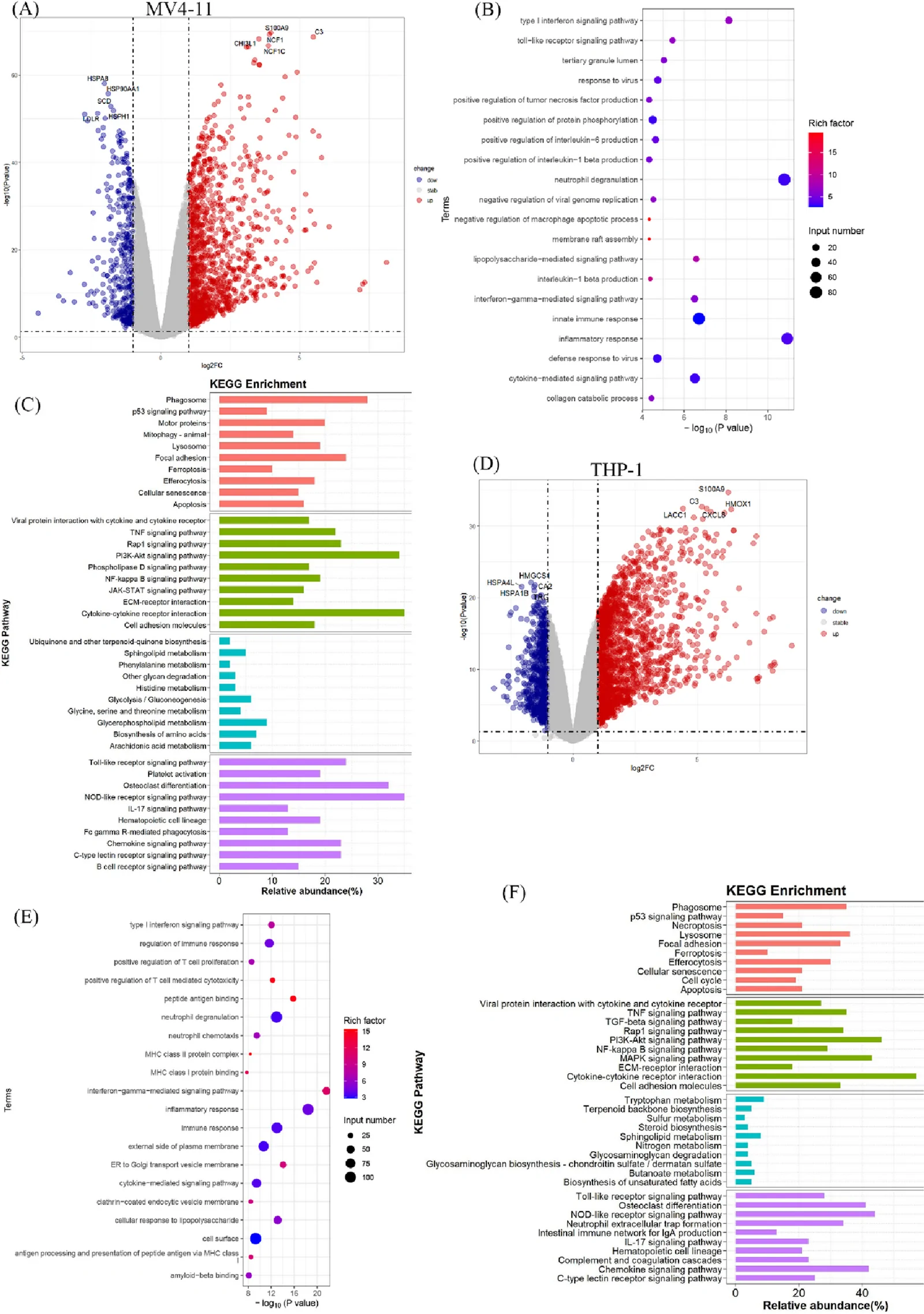
Transcriptome analysis of K-Env KO and mock group cells. **A**, Volcano plots of gene expression changes in MV4-11 K-Env KO and mock groups. **B**–**C** Gene ontology (GO) and Kyoto encyclopedia of genes and genomes (KEGG) analysis of differentially expressed genes (DEGs) in MV4-11 K-Env KO and mock cells. **D** Volcano plots of gene expression changes in THP-1 K-Env KO and mock groups. **E**–**F** GO and KEGG analysis of DEGs in THP-1 K-Env KO and mock cells

To elucidate the molecular mechanisms by which HERV-K102 Env contributes to AML pathogenesis, we performed transcriptome profiling of K-Env KO MV4-11 and THP-1 cells alongside mock controls. Comparative analyses revealed widespread transcriptomic alterations following K-Env ablation. In MV4-11 cells, K-Env KO resulted in 1762 differentially expressed genes (DEGs), with 1,351 upregulated and 411 downregulated genes (Fig. 3A). Among the most significantly upregulated genes, S100A9, C3, CHI3L1, NCF1, and NCF1C are key mediators of inflammation and immune responses. GO enrichment analysis showed that these DEGs were strongly associated with the inflammatory response, neutrophil degranulation, and cytokine-mediated signaling pathways (Fig. 3B). KEGG pathway analysis further indicated robust activation of the NOD-like receptor, PI3K-Akt, and NF-κB signaling pathways (Fig. 3C), suggesting strong engagement of innate immune and survival-related molecular networks. Similarly, in THP-1 cells, K-Env depletion significantly altered 2,421 genes, with 1,706 and 715 upregulated and downregulated genes, respectively (Fig. 3D). Notably, S100A9, C3, HMOX1, CXCL8, and LACC1 were the most upregulated genes, reinforcing the pattern of innate immune activation. GO analysis revealed enrichment in the inflammatory response, immune activation, and neutrophil degranulation (Fig. 3E). KEGG analysis also confirmed significant involvement of the NOD-like receptor, PI3K-Akt, NF-κB, and cytokine–cytokine receptor interaction pathways (Fig. 3F). Collectively, these findings indicate that HERV-K102 Env ablation triggers a strong inflammatory response and activates key immunomodulatory and survival signaling pathways, suggesting that K-Env may suppress immune recognition and help maintain cellular homeostasis in AML.

### Knockout of K-Env induces pyroptosis via activation of the S100A9/NOD-like receptor signaling pathway

**Fig. 4:**
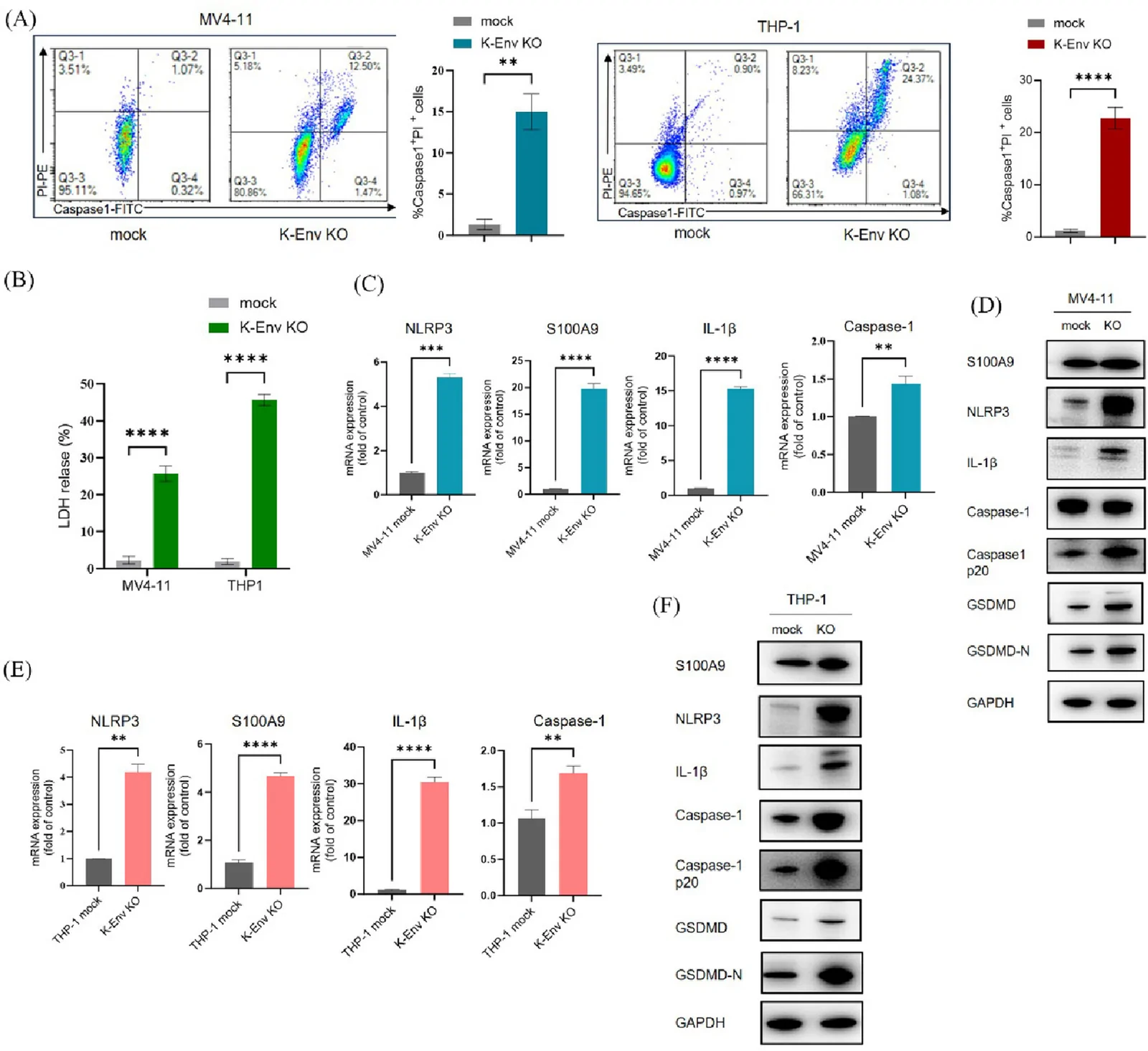
K-Env KO mediates pyroptosis by upregulating S100A9 and the NOD-like receptor signaling pathway. **A** The percentage of Caspase-1^+^PI^+^ cells was detected by flow cytometry. **B** Increased release of lactate dehydrogenase (LDH) was observed in the K-Env KO and mock groups in AML cells. **C** qRT-PCR analysis of NLRP3, S100A9, IL-1β, and caspase 1 mRNA levels in MV4-11 mock and K-Env KO cells. **D** Western blot analysis of pyroptosis-related proteins in MV4-11 mock and K-Env KO cells. **E** qRT-PCR analysis of NLRP3, S100A9, IL-1β, caspase-1 P20, and GSDMD mRNA levels in THP-1 mock and K-Env KO cells. **F** Western blot analysis of pyroptosis-related proteins in THP-1 mock and K-Env KO cells

Given that K-Env knockout upregulated the genes involved in inflammatory regulation, we investigated whether K-Env knockout induces pyroptosis in leukemia cells. Caspase-1 is a key protease that initiates pyroptosis. The proportion of caspase1^+^PI^+^ cells was significantly higher in the K-Env KO mice (Fig. 4A). On day 6 after K-Env knockout, we measured LDH levels. The results showed that cells in the knockout group released more LDH (Fig. 4B). To further elucidate potential molecular mechanisms, we conducted additional tests on molecules related to pyroptosis. The mRNA levels of S100A9, caspase-1, IL-1β, NLRP3, and GSDMD were significantly elevated in the MV4-11 K-Env KO group (Fig. 4C). Consistent with these findings, western blot analysis confirmed increased protein expression of S100A9, caspase-1 P20, IL-1β, GSDMD-N, and NLRP3 in the knockout group (Fig. 4D). Similar results were observed in THP-1 cells (Fig. 4E,F). Here, we hypothesized that knockout of K-Env may cause pyroptosis by upregulating S100A9 and the NOD-like receptor signaling pathway. During this process, the expressions of caspase-1, IL-1β, GSDMD, and NLRP3 also increase.

### K-Env silencing suppresses leukemia growth in mouse models

**Fig. 5:**
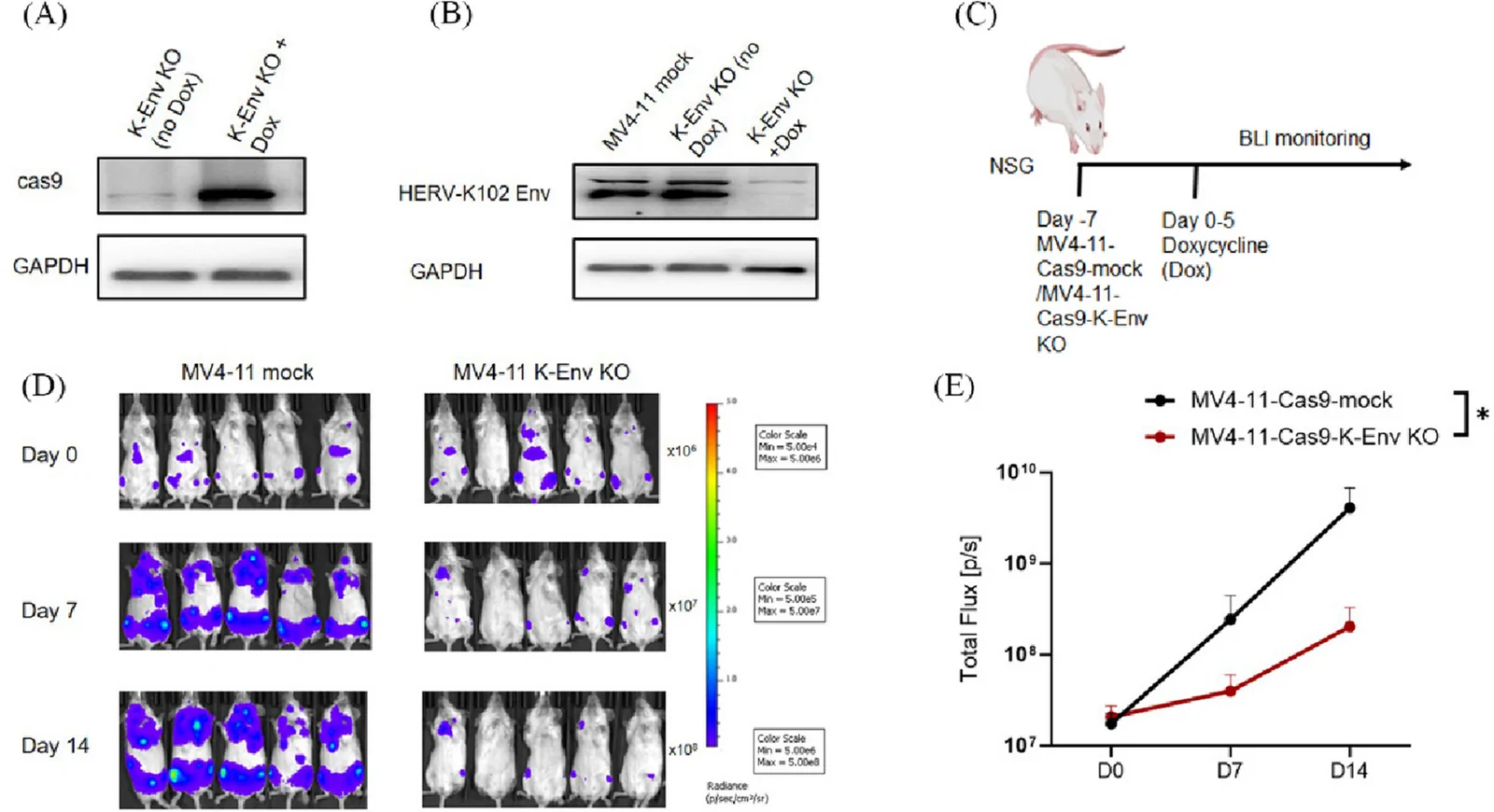
Knockout of K-Env inhibits the proliferation of AML cells in vivo. **A** Constructed MV4-11 cas9 cells, also showing that the cells could express cas9 protein after doxycycline (Dox) induction. **B** K-Env knocked out by western blot after induction with Dox. **C** Experimental scheme of the in vivo evaluation of MV4-11 and MV4-11 K-Env KO xenograft model. **D** Bioluminescent imaging analysis in tumor-bearing mice (*n* = 5). **E** The average radiance measured over the course of 14 days as an indication of tumor growth in vivo

To further evaluate the in vivo efficacy of K-Env knockout, which was shown to inhibit cell proliferation, we established an inducible knockout model using the Tet-On system. First, the MV4-11 cell line was transduced with the pcw-Cas9 lentivirus. Upon induction with 2 μg/mL Dox, Cas9 protein expression was significantly and stably upregulated (Fig. 5A). Subsequently, plx-sgRNA lentivirus was transduced into MV4-11-Cas9 cells. Western blot analysis confirmed that K-Env protein levels were markedly reduced following Dox treatment (Fig. 5B). Consistent with this, the MTT assay performed on day 5 after Dox induction showed inhibited proliferation in cells in the Dox-induced knockout group only (Figure S2). For in vivo validation, MV4-1-Cas9-mock and MV4-11-Cas9-K-Env KO cells were injected into mice via the tail vein on day 7 (Fig. 5D). Tumor implantation was observed through fluorescence imaging on day 0, and the results showed no significant difference in tumor burden between the two groups. Dox was used to induce Cas9 expression in knockout K-Env. Subsequently, the growth of tumor cells in the mice was monitored using fluorescence imaging. The results showed that tumor signals in the KO group were significantly lower than those in the control group (Fig. 5E,F). These results demonstrate that induced silencing of K-Env effectively suppressed AML cell proliferation in vivo.

## Discussion

HERVs are the remnants of ancient retroviral infections that integrated into the human genome. HERV-K is typically transcriptionally silenced in normal cells, but is reactivated during malignant transformation [6, 23]. Abnormal HERV-K Env expression is associated with cancer cell proliferation and tumorigenesis [10, 12] as well as autoimmune diseases such as rheumatoid arthritis (RA) and systemic lupus erythematosus (SLE) [24, 25]. Previous studies showed that HERV-K102 Env proteins are specifically present in the sera of cancer patients [26]. Our study further confirmed the upregulation of K-Env at both mRNA and protein levels using databases and AML cell lines. However, the biological function of HERV-K102 in AML remains unclear. Studies have also shown that HERV-K119 Env knockout suppresses the proliferation, migration, and invasion of colorectal and ovarian cancer cells [27], whereas HERV-K Env knockdown inhibits the proliferation and metastasis of pancreatic cancer [28]. Based on these findings, we hypothesized that the HERV-K102 envelope exerts similar oncogenic effects in AML. We demonstrated that CRISPR-Cas9-mediated knockout of K-Env significantly inhibited proliferation and promoted apoptosis in AML cell lines. Notably, these phenotypes were consistent across multiple cell lines, highlighting the broad functional relevance of K-Env in AML.

In addition to apoptosis, we provided the first evidence that K-Env knockout induces pyroptosis, a highly inflammatory type of programmed cell death, via the S100A9/NOD-like receptor signaling pathway. RNA-Seq identified S100A9 as the most significantly upregulated gene after K-Env ablation. S100A9 promotes inflammation through Toll-like receptor 4 (TLR4) and receptor for advanced glycation end product (RAGE) signaling in both immune and tumor cells [29] and has been implicated in inflammation-driven cancers [30, 31]. Consistent with these reports, K-Env knockout not only induced apoptosis and suppressed proliferation but also enriched inflammatory pathways. We showed that K-Env knockout activated this cascade through S100A9 upregulation and NLRP3 inflammasome activation. These findings revealed a previously unrecognized role for K-Env in suppressing pyroptosis in AML. Ablation-induced inflammatory cell death reveals a novel mechanism with potential therapeutic implications.

However, this study has several shortcomings. Firstly, HERV-K is classified into two types: type-1, which has a 292 bp deletion in the 5' region of env, and type-2, which has an intact envelope gene [32]. The primers used in this study were based on a universal HERV-K sequence, which made it impossible to specifically target HERV-K102 at the mRNA level. Ideally, site-specific methods such as site-specific qPCR or targeted sequencing should be used. Therefore, protein level verification of this study is of crucial importance. Secondly, markers such as LDH release, caspase-1 activation, and GSDMD cleavage may have a certain degree of overlap with cell apoptosis or secondary necrosis. This requires functional verification using specific inhibitors to effectively distinguish between pyroptosis, apoptosis, necrosis, and other general cytotoxic deaths.

AML is characterized by high heterogeneity and poor prognosis. DNA demethylating drugs (HMAs) are often used as the "cornerstone,” treatment and are combined with drugs with different mechanisms of action, such as venetoclax, to enhance therapeutic efficacy. HMAs inhibit DNA methyltransferases, causing the demethylation of HERV promoters (especially the LTR region) and directly activating their transcription [33]. On the one hand, this provides more abundant targets for antibody therapy against HERV-K102. On the other hand, by activating "virus mimicry" immune responses, it may create a more favorable immune microenvironment. However, the immunosuppressive effects of HMAs and risk of tolerance caused by excessive HERV expression must be carefully evaluated using a combined strategy. Furthermore, there may be risks associated with the induction of AML cell pyroptosis by knocking out K-Env. The pyroptosis of massive cells may trigger a systemic, highly inflammatory state [34], such as a cytokine storm, leading to organ damage. Secondly, the HERV-K102 Env protein itself (especially the transmembrane subunit) exhibits immunosuppressive activity [26]. Thus, the knockout of K-Env may cause an imbalance in the immune environment.

In summary, our study established that HERV-K102 is highly expressed in AML, which may be related to the occurrence and development of the disease. HERV-K102 envelope (K-Env) is a promising diagnostic and therapeutic target, potentially enabling the development of new immunotherapy-based strategies for AML treatment.

## Supplementary Information

Supplementary Material 1: Figure S1. The relationship between HERV-K102 expression and prognosis of AML patients. The expression level of HERV-K102 is divided into two categories based on the median.

Supplementary Material 2: Figure S2. Inhibition of cell proliferation after K-Env knocked out by Dox induction. Cell viability was determined by MTT assay on day 5 after Dox induction.

## Data Availability

Requests for resources and reagents can be addressed directly to the corresponding authors. All data generated or analyzed during this study that are not included in this published article and its supplementary information are available from the corresponding author on reasonable request.

## References

[1] Bannert N, Kurth R. The evolutionary dynamics of human endogenous retroviral families. Annu Rev Genomics Hum Genet. 2006;7:149–73. 10.1146/annurev.genom.7.080505.115700. 16722807

[2] Subramanian RP, Wildschutte JH, Russo C, Coffin JM. Identification, characterization, and comparative genomic distribution of the HERV-K (HML-2) group of human endogenous retroviruses. Retrovirology. 2011;8:90. 10.1186/1742-4690-8-90. PMC3228705 22067224

[3] Dewannieux M, et al. Identification of an infectious progenitor for the multiple-copy HERV-K human endogenous retroelements. Genome Res. 2006;16:1548–56. 10.1101/gr.5565706. PMC1665638 17077319

[4] Boller K, et al. Human endogenous retrovirus HERV-K113 is capable of producing intact viral particles. J Gen Virol. 2008;89:567–72. 10.1099/vir.0.83534-0. 18198388

[5] Downey RF, et al. Human endogenous retrovirus K and cancer: innocent bystander or tumorigenic accomplice? Int J Cancer. 2015;137:1249–57. 10.1002/ijc.29003. PMC6264888 24890612

[6] Greenig M. HERVs, immunity, and autoimmunity: understanding the connection. PeerJ. 2019;7:e6711. 10.7717/peerj.6711. PMC6452852 30984482

[7] Römer C. Viruses and endogenous retroviruses as roots for neuroinflammation and neurodegenerative diseases. Front Neurosci. 2021;15:648629. 10.3389/fnins.2021.648629. PMC7994506 33776642

[8] Liu X, et al. Resurrection of endogenous retroviruses during aging reinforces senescence. Cell. 2023;186:287-304.e226. 10.1016/j.cell.2022.12.017. 36610399

[9] Burns KH. Transposable elements in cancer. Nat Rev Cancer. 2017;17:415–24. 10.1038/nrc.2017.35. 28642606

[10] Contreras-Galindo R, et al. Human endogenous retrovirus K (HML-2) elements in the plasma of people with lymphoma and breast cancer. J Virol. 2008;82:9329–36. 10.1128/jvi.00646-08. PMC2546968 18632860

[11] Wang-Johanning F, et al. Human endogenous retrovirus K triggers an antigen-specific immune response in breast cancer patients. Cancer Res. 2008;68:5869–77. 10.1158/0008-5472.Can-07-6838. PMC5802396 18632641

[12] Wang-Johanning F, et al. Expression of multiple human endogenous retrovirus surface envelope proteins in ovarian cancer. Int J Cancer. 2007;120:81–90. 10.1002/ijc.22256. 17013901

[13] Maliniemi P, et al. Expression of human endogenous retrovirus-w including syncytin-1 in cutaneous T-cell lymphoma. PLoS ONE. 2013;8:e76281. 10.1371/journal.pone.0076281. PMC3788054 24098463

[14] Stengel S, Fiebig U, Kurth R, Denner J. Regulation of human endogenous retrovirus-K expression in melanomas by CpG methylation. Genes Chromosomes Cancer. 2010;49:401–11. 10.1002/gcc.20751. 20095041

[15] Januszkiewicz-Lewandowska D, et al. Env gene expression of human endogenous retrovirus-k and human endogenous retrovirus-w in childhood acute leukemia cells. Acta Haematol. 2013;129:232–7. 10.1159/000345407. 23328642

[16] Curty G, et al. Human endogenous retrovirus K in cancer: A potential biomarker and immunotherapeutic target. Viruses. 2020;12. 10.3390/v12070726. PMC7412025 32640516

[17] Hsu SK, et al. Inflammation-related pyroptosis, a novel programmed cell death pathway, and its crosstalk with immune therapy in cancer treatment. Theranostics. 2021;11:8813–35. 10.7150/thno.62521. PMC8419056 34522213

[18] Wei X, et al. Role of pyroptosis in inflammation and cancer. Cell Mol Immunol. 2022;19:971–92. 10.1038/s41423-022-00905-x. PMC9376585 35970871

[19] Wang S, et al. S100A8/A9 in inflammation. Front Immunol. 2018;9:1298. 10.3389/fimmu.2018.01298. PMC6004386 29942307

[20] Basiorka AA, et al. The NLRP3 inflammasome functions as a driver of the myelodysplastic syndrome phenotype. Blood. 2016;128:2960–75. 10.1182/blood-2016-07-730556. PMC5179338 27737891

[21] Zhang Q, et al. EZH2 inhibition induces pyroptosis via RHA-mediated S100A9 overexpression in myelodysplastic syndromes. Exp Hematol Oncol. 2025;14:9. 10.1186/s40164-025-00600-3. PMC11780917 39881418

[22] Li M, et al. HERV-K TM subunit elicits CD8(+) T cell anergy and tumor immune evasion via targeting CD3 coreceptor ε in AML and PDAC. Adv Sci (Weinh). 2026;e17432. 10.1002/advs.202417432. PMC12767058 40801282

[23] Fu B, Ma H, Liu D. Functions and regulation of endogenous retrovirus elements during zygotic genome activation: Implications for improving somatic cell nuclear transfer efficiency. Biomolecules. 2021;11. 10.3390/biom11060829. PMC8229993 34199637

[24] Tokuyama M, et al. Antibodies against human endogenous retrovirus K102 envelope activate neutrophils in systemic lupus erythematosus. J Exp Med. 2021;218. 10.1084/jem.20191766. PMC8144942 34019642

[25] Laine A, et al. Expression of envelope protein encoded by endogenous retrovirus K102 in rheumatoid arthritis neutrophils. Microorganisms. 2023;11. 10.3390/microorganisms11051310. PMC10223813 37317284

[26] Gong Q, Xu R. Subtype-specific human endogenous retrovirus K102 envelope protein is a novel serum immunosuppressive biomarker of cancer. Front Immunol. 2024;15:1533740. 10.3389/fimmu.2024.1533740. PMC11754298 39850893

[27] Ko EJ, et al. Human endogenous retrovirus (HERV)-K env gene knockout affects tumorigenic characteristics of nupr1 gene in DLD-1 colorectal cancer cells. Int J Mol Sci. 2021;22. 10.3390/ijms22083941. PMC8070087 33920455

[28] Li M, et al. Downregulation of human endogenous retrovirus type K (HERV-K) viral env RNA in pancreatic cancer cells decreases cell proliferation and tumor growth. Clin Cancer Res. 2017;23:5892–911. 10.1158/1078-0432.Ccr-17-0001. PMC5777511 28679769

[29] Leach ST, et al. Serum and mucosal S100 proteins, calprotectin (S100A8/S100A9) and S100A12, are elevated at diagnosis in children with inflammatory bowel disease. Scand J Gastroenterol. 2007;42:1321–31. 10.1080/00365520701416709. 17852869

[30] Bresnick AR, Weber DJ, Zimmer DB. S100 proteins in cancer. Nat Rev Cancer. 2015;15:96–109. 10.1038/nrc3893. PMC4369764 25614008

[31] Funk S, et al. High S100A8 and S100A12 protein expression is a favorable prognostic factor for survival of oropharyngeal squamous cell carcinoma. Int J Cancer. 2015;136:2037–46. 10.1002/ijc.29262. 25302747

[32] Shin W, Mun S, Han K. Human endogenous retrovirus-K (HML-2)-related genetic variation: Human genome diversity and disease. Genes. 2023;14. 10.3390/genes14122150. PMC10742618 38136972

[33] Daskalakis M, et al. Reactivation of endogenous retroviral elements via treatment with DNMT- and HDAC-inhibitors. Cell Cycle. 2018;17:811–22. 10.1080/15384101.2018.1442623. PMC6056222 29633898

[34] Vasudevan SO, Behl B, Rathinam VA. Pyroptosis-induced inflammation and tissue damage. Semin Immunol. 2023;69:101781. 10.1016/j.smim.2023.101781. PMC10598759 37352727

