# Supplementary figures and images for: Targeting the HERV-K102 envelope elicits pyroptosis and represents a novel therapeutic strategy for acute myeloid leukemia
Source: Blood Res. 2026 Apr 14;61(1):23. doi: 10.1007/s44313-026-00134-5 (PMC13184007; doi:10.1007/s44313-026-00134-5)

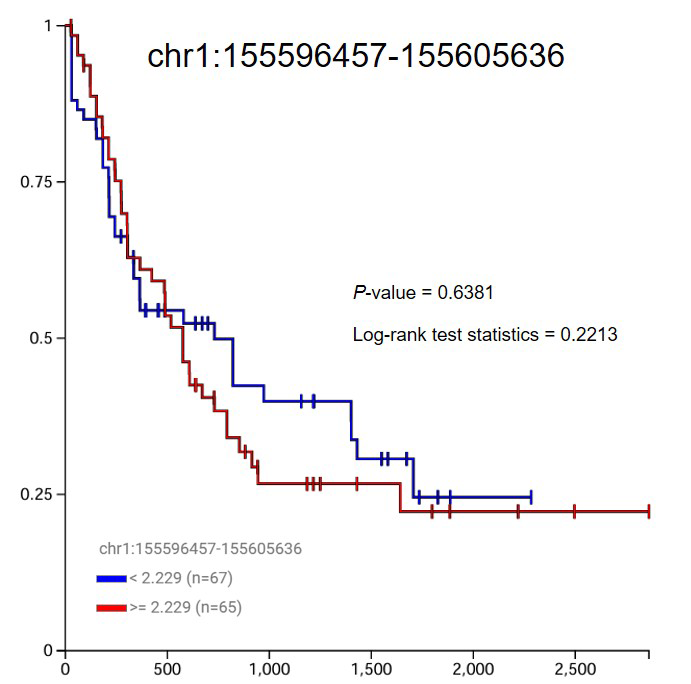

Supplement: Supplementary file 1 — Supplementary Material 1: Figure S1. The relationship between HERV-K102 expression and prognosis of AML patients. The expression level of HERV-K102 is divided into two categories based on the median. [file 44313_2026_134_MOESM1_ESM.tif]
